# Forestier’s disease: a cause of dysphagia to recall

**DOI:** 10.1590/S1679-45082014AI2659

**Published:** 2014

**Authors:** Francisco Otavio Camargo Pereira, Flavio Ramalho Romero, Kleber Carlos Azevedo, Ismael Augusto Silva Lombardi, Priscila Watson Ribeiro, Roberto Colichio Gabarra, Marco Antonio Zanini

**Affiliations:** 1 Universidade Estadual Paulista “Júlio de Mesquita Filho”, Botucatu, SP, Brazil.

**Figure:**
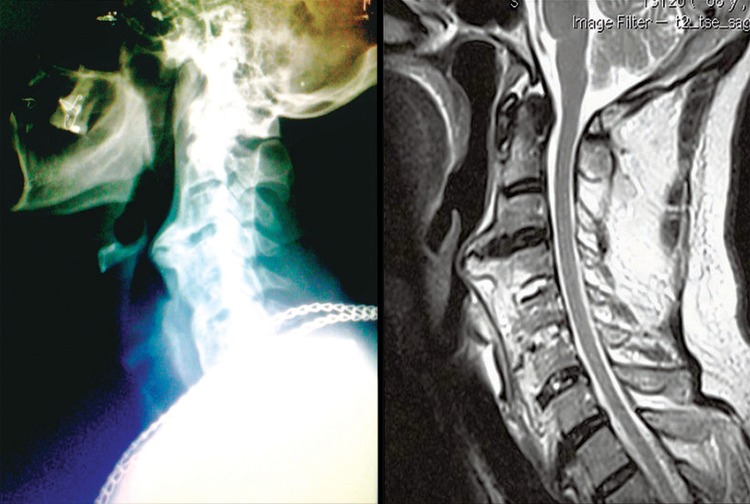
Lateral cervical X-ray and T2-weighted sagittal magnetic resonance. We observed an extensive calcification in front of vertebral bodies of C2 to C6 in topography of anterior longitudinal ligament. Esophagus and airways present a posterior compromising, which determine a stenosed segment that difficult orotracheal intubation at time of surgical procedure

A 68-year-old man diagnosed with Chagas disease, hypertension and type 2 diabetes mellitus was followed up at a quarterly hospital in the specialties of otolaryngology and gastroenterology because of a chronic clinical feature of dysphagia and odynophagia for solid foods, and a non-confirmed diagnosis hypothesis of chagasic megaesophagus.

During complementary investigation he was submitted to lateral simple cervical radiograph that revealed an extrinsic compression of the laryngeal esophagus secondary to exuberant osteophyte in C3-C4 level. Radiography showed involvement of adjacent levels (C2-C3, C4-C5 and C5-C6), ossification of anterior longitudinal ligament and preservation of disc spaces height. Such findings, along with the absence of radiologic compromising of sacroiliac joints, correspond to Resnick criteria^(1)^ found in the diffuse idiopathic skeletal hyperostosis (DISH) described by J. Forestier in 1950.^(2)^

The Forestier’s disease is characterized by bone proliferation at sites of insertion of ligaments and tendons^(3,4)^ (enthesopathy). Most of patients are asymptomatic and the disease is discovered incidentally or when other symptoms are investigated. In addition, the disease often affects men over 50 years old and presents a correlation with diabetes.^(5)^ The patient in this report had a hard-to-control hyperglycemia despite optimal clinical treatment.

Main differential diagnoses^(6)^ are ankylosing spondylitis that affects younger individuals and is more symptomatic and spondyloarthritis that traction osteophytes occurs and no damage in anterior longitudinal ligament is seen.

In general, DISH does not present a specific treatment because it evolves slowly and most of patients are asymptomatic.^(7)^ Drug treatment, change in food habits, use of muscle relaxants associated with physiotherapy is a good option for cases of mild to moderate symptomatology. In our case, we had chosen surgical treatment because of the severe symptomatology and compromising of patient’s quality of life. An osteophytectomy via the anterolateral cervical access was performed without intercurrences, and the patient showed expressive improvement of previous symptomatology and was discharged 48 hours after the procedure.
