# Supplementary material for: Forestier’s disease: a cause of dysphagia to recall
Source: Einstein (Sao Paulo). 2014 Jul-Sep;12(3):380–1. [Article in Portuguese] doi: 10.1590/S1679-45082014AI2659 (PMC4872955; doi:10.1590/S1679-45082014AI2659)
Supplement: Translation

# Translation: Doença de Forestier: uma causa de disfagia a ser lembrada

**Authors:** Francisco Otavio Camargo Pereira, Flavio Ramalho Romero, Kleber Carlos Azevedo, Ismael Augusto Silva Lombardi, Priscila Watson Ribeiro, Roberto Colichio Gabarra, Marco Antonio Zanini

**Affiliations:** 1 Universidade Estadual Paulista “Júlio de Mesquita Filho”, Botucatu, SP, Brasil.

**Figure:** Raio X cervical em perfil e ressonância magnética sagital ponderada em T2. Observamos extensa calcificação em frente aos corpos vertebrais de C2 a C6 na topografia do ligamento longitudinal anterior. O esôfago e a via aérea apresentam-se comprimidos posteriormente, determinando um segmento estenosado, o que dificultou a intubação orotraqueal na ocasião do procedimento cirúrgico

Paciente do gênero masculino, 68 anos, portador de doença de Chagas, hipertensão arterial e *diabetes mellitus* tipo 2. Acompanhado em hospital quaternário pelas especialidades Otorrinolaringologia e Gastroenterologia devido à quadro crônico de disfagia e odinofagia para alimentos sólidos, com a hipótese diagnóstica não confirmada de megaesôfago chagásico.

Durante investigação complementar foi submetido à radiografia cervical simples em perfil, a qual evidenciou compressão extrínseca esôfago-laríngea secundária à osteófito exuberante, no nível C3-C4. Na radiografia, puderam ser observados ainda acometimento dos níveis adjacentes (C2-C3, C4-C5 e C5-C6), ossificação do ligamento longitudinal anterior e preservação da altura dos espaços discais. Tais achados, somados à ausência de comprometimento radiológico das articulações sacroilíacas, correspondem aos critérios de Resnick^(1)^ encontrados na hiperostose esquelética idiopática difusa (DISH, sigla do inglês *diffuse idiopathic skeletal hyperostosis*), descrita primeiramente em 1950 por J. Forestier.^(2)^

A doença de Forestier caracteriza-se por proliferação óssea em locais de inserção dos ligamentos e tendões^(3,4)^ (entesopatia), sendo que a maior parte dos pacientes é assintomática e a doença é descoberta de forma incidental ou em investigação de outros sintomas. Acomete com maior frequência indivíduos do gênero masculino acima dos 50 anos, sendo observada uma correlação com o diabetes.^(5)^ O paciente em questão apresentava hiperglicemia de difícil controle, a despeito de tratamento clínico otimizado.

Os principais diagnósticos diferenciais^(6)^ são a espondilite anquilosante, que acomete indivíduos mais jovens e é mais sintomática, e a espondilodiscartrose, na qual ocorrem osteófitos de tração e não há comprometimento do ligamento longitudinal anterior.

De modo geral a DISH não apresenta tratamento específico, uma vez que a evolução é lenta, e os pacientes são em sua maioria assintomáticos.^(7)^ O tratamento clínico medicamentoso, composto por mudança de hábitos alimentares e pelo uso de relaxantes musculares associado à fisioterapia, é uma boa opção para os casos de sintomatologia leve a moderada. Em nosso caso, optamos pelo tratamento cirúrgico devido à sintomatologia grave e ao comprometimento da qualidade de vida do paciente. A osteofitectomia cirúrgica por acesso cervical anterolateral foi realizada sem intercorrências, e o paciente apresentou melhora expressiva da sintomatologia prévia, recebendo alta hospitalar 48 horas após o procedimento.
